# Editorial: Nutrient Sensing: The Constant in All Other Things

**DOI:** 10.3389/fendo.2021.705640

**Published:** 2021-06-15

**Authors:** Anne-Sophie Vercoutter-Edouart, Chad Slawson

**Affiliations:** ^1^ Structural and Functional Glycobiology Unit, University of Lille, Lille, France; ^2^ Department of Biochemistry and Molecular Biology, University of Kansas Medical Center, Kansas City, KS, United States

**Keywords:** nutrients, nutrient flux, post-translational modifications, signaling, metabolism

The ability of cells to sense nutrients is a constant in all things. Nutrient availability controls all aspects of cellular function including when to grow, when to divide, how to move, what metabolites to process, what to communicate, and what genes to turn on or turn off. Understanding the cellular mechanisms that sense nutrient flux and control cellular response to nutrient flux is therefore vital to understanding the biochemistry and physiology of a cell.

Often, nutrients are the sensor, post-translational modifications (PTM) tend to be metabolites derived from nutrients. Nutrient availability dictates the amount of the metabolite available as a substrate for enzymes to modify a resulting protein and regulate cellular function. Too little of the metabolite will activate pro-survival cellular processes and influence metabolic pathways to provide the missing nutrient if possible; while too much metabolite can lead to cellular disruption, non-enzymatic protein modifications, and aberrant enzymatic function. These changes have huge consequences for the health of the cell or the entire organism. As the complexity of the cell increases the integration of nutrient signals increases with exponential growth in post-translation modifications from ancient modifications such as acetylation to evolutional new PTMs like tyrosine phosphorylation and O-GlcNAcylation. Accordingly, cell nutrient sensing is of paramount significance and value.

The focus of this Research Topic of Frontiers in Endocrinology is to gain a greater understanding of the mechanisms through which cells sense nutrient flux to regulate cellular function. 

The articles featured in this Research Topic link nutrient sensing post-translational modifications to cancer cell stem state, how glucose controls DNA methylation, explore the role of AMP-activated protein kinase in cell growth, and how immune cell function is controlled by O-GlcNAcylation. Together, these original articles and reviews shed light on the potential cellular mechanisms that sense nutrients.

Robles-Flores et al. do a deep dive into how nutrient sensing is key to controlling the function of cancer stem cells with a focus on three inter-connected nutrient sensing pathways: AMP-activated protein kinase (AMPK), mammalian target of rapamycin (mTOR), and the hexosamine biosynthetic pathway. Siao et al. further explore the function of AMPK in preadipocytes after extracellular stimulation by endothelins. Cai et al. investigate how nutrient excess is translated into DNA methylation changes. Lastly, Qiang et al. describe the emerging field of immune metabolism and the role of O-GlcNAcylation in controlling cell function.

In summary, this Research Topic presents an ever-evolving understanding of the role of nutrients in dictating cellular function. Importantly, these articles raise new questions as to how the crosstalk of multiple nutrient sensing pathways might regulate a response to a specific nutrient or cause a metabolite to be used for a different cellular process. As our understanding of disease expands, the contribution of nutrient sensing is ever constant and central to this process.

## Author Contributions

Together, CS and A-SV-E wrote the editorial. All authors contributed to the article and approved the submitted version.

## Conflict of Interest

The authors declare that the research was conducted in the absence of any commercial or financial relationships that could be construed as a potential conflict of interest.

